# Reduced cortical cholinergic innervation measured using [^18^F]-FEOBV PET imaging correlates with cognitive decline in mild cognitive impairment

**DOI:** 10.1016/j.nicl.2022.102992

**Published:** 2022-03-24

**Authors:** Ying Xia, Eamonn Eeles, Jurgen Fripp, Donna Pinsker, Paul Thomas, Melissa Latter, Vincent Doré, Amir Fazlollahi, Pierrick Bourgeat, Victor L. Villemagne, Elizabeth J. Coulson, Stephen Rose

**Affiliations:** a The Australian e-Health Research Centre, CSIRO Health and Biosecurity, Brisbane, QLD, Australia; b Internal Medicine Service, The Prince Charles Hospital, Brisbane, QLD, Australia; c School of Medicine, Northside Clinical School, The Prince Charles Hospital, Brisbane, QLD, Australia; d Dementia & Neuro Mental Health Research Unit, UQCCR, Royal Brisbane and Women’s Hospital, Brisbane, QLD, Australia; e School of Psychology, The University of Queensland, Brisbane, QLD, Australia; f Royal Brisbane and Women’s Hospital, Brisbane, QLD, Australia; g Austin Health, Melbourne, VIC, Australia; h Queensland Brain Institute, The University of Queensland, Brisbane, QLD, Australia; i Department of Psychiatry, University of Pittsburgh, Pittsburgh, PA, USA; j School of Biomedical Sciences, The University of Queensland, Brisbane, QLD, Australia

**Keywords:** Ach, acetylcholine, BF, basal forebrain, CU, cognitively unimpaired, FBB, florbetaben, FEOBV, fluoroethoxybenzovesamicol, GM, grey matter, MCI, mild cognitive impairment, MPRAGE, magnetization-prepared rapid gradient echo, NBM, nucleus basalis of Meynert, SUVR, standardized uptake value ratio, TFCE, threshold free cluster enhancement, TIV, total intracranial volume, UTE, ultrashort echo-time, VAChT, vesicular acetylcholine transporter, WM, white matter, Basal forebrain, Cholinergic system, FEOBV, Mild cognitive impairment, PET imaging, MRI

## Abstract

Dysfunction of the cholinergic basal forebrain (BF) neurotransmitter system, including cholinergic axon denervation of the cortex, plays an important role in cognitive decline and dementia. A validated method to directly quantify cortical cholinergic terminal integrity enables exploration of the involvement of this system in diverse cognitive profiles associated with dementia, particularly at a prodromal stage. In this study, we used the radiotracer [^18^F]-fluoroethoxybenzovesamicol (FEOBV) as a direct measure of cholinergic terminal integrity and investigated its value for the assessment of cholinergic denervation in the cortex and associated cognitive deficits. Eighteen participants (8 with mild cognitive impairment (MCI) and 10 cognitively unimpaired controls) underwent neuropsychological assessment and brain imaging using FEOBV and [^18^F]-florbetaben for amyloid-β imaging. The MCI group showed a significant global reduction of FEOBV retention in the cortex and in the parietal and occipital cortices specifically compared to the control group. The global cortical FEOBV retention of all participants positively correlated with the BF, hippocampus and grey matter volumes, but no association was found between the global FEOBV retention and amyloid-β status. Topographic profiles from voxel-wise analysis of FEOBV images revealed significant positive correlations with the cognitive domains associated with the underlying cortical areas. Overlapping profiles of decreased FEOBV were identified in correlation with impairment in executive function, attention and language, which covered the anterior cingulate gyrus, olfactory cortex, calcarine cortex, middle temporal gyrus and caudate nucleus. However, the absence of cortical atrophy in these areas suggested that reduced cholinergic terminal integrity in the cortex is an important factor underlying the observed cognitive decline in early dementia. Our results provide support for the utility and validity of FEOBV PET for quantitative assessment of region-specific cholinergic terminal integrity that could potentially be used for early detection of cholinergic dysfunction in dementia following further validation in larger cohorts.

## Highlights

• Topographic FEOBV binding correlates with domain-specific cognitive performance.
• Global and regional reductions in cholinergic innervation are observed in MCI.
• Global FEOBV SUVR is associated with basal forebrain and hippocampal volumes.
• Our results provide proof of concept for FEOBV PET to assess cholinergic terminal integrity.

## 1 Introduction

Dysfunction of the cholinergic basal forebrain (BF) neurotransmitter system is known to play an important role in cognitive decline in neurodegenerative disorders such as Alzheimer’s disease (AD) (Bohnen et al., 2018). The cholinergic projection system originating from the BF provides the neurotransmitter acetylcholine (ACh) to the cerebral cortex and hippocampus, and directly influences critical cognitive functions, particularly memory and attention processing (Ballinger et al., 2016, Prado et al., 2017, Schliebs and Arendt, 2011). Increasing evidence has suggested that a complex interaction exists between the cholinergic system and pathological features of AD, whereby amyloid-β (Aβ) can be toxic to cholinergic neurons and cholinergic dysfunction may promote Aβ deposition and tau pathology in ways that contribute to cognitive impairment (Hampel et al., 2018, Ramos-Rodriguez et al., 2013, Schliebs, 2005). Loss of cortical cholinergic innervation is also found *in vivo* in patients with mild cognitive impairment (MCI), representing the prodromal stage of AD (Haense et al., 2012). Moreover, the correlation of the BF cholinergic neuron loss and cortical thinning in the projecting regions was confirmed in patients affected by MCI (Kilimann et al., 2017). However, the patterns of cortical cholinergic denervation in association with different cognitive profiles have not yet been fully investigated, particularly at early stages of the disease, which would be beneficial for identifying vulnerable brain structures involved in different cognitive deficits and potentially facilitate earlier diagnosis of dementia.

Currently, there is no robust and sensitive tool for *in vivo* quantification of cholinergic dysfunction in the brain. Using magnetic resonance imaging (MRI), volumetric changes in the BF can be detected and have been used as a surrogate marker of cholinergic degeneration in ageing and dementia (Grothe et al., 2018, Grothe et al., 2014, Hall et al., 2008, Lammers et al., 2018, Teipel et al., 2005). Recent studies have demonstrated a robust association between BF atrophy and cognitive deficits in AD (Grothe et al., 2016). Significant BF volume loss was detected at preclinical (Scheef et al., 2019) and early stages of AD (Cavedo et al., 2017, Grothe et al., 2012). BF atrophy correlated with Aβ accumulation in amnestic MCI as well as AD (Fernández-Cabello et al., 2020, Kerbler et al., 2015, Teipel et al., 2014), and more recently, volume loss of the nucleus basalis of Meynert (NBM) was associated with tau pathology in individuals at risk for AD (Cantero et al., 2020). Although these findings regarding BF atrophy provide encouraging support for the cholinergic hypothesis of dementia, unambiguous detection of atrophic changes by MRI is only likely to be possible after significant neuronal loss has already occurred.

Direct assessment of the cholinergic system *in vivo* is the preferred method of providing definitive information regarding early deficiency in cholinergic neurotransmission and innervation prior to frank neuronal and volume loss. Fluorine-18 fluoroethoxybenzovesamicol ([^18^F]-FEOBV) is one of the most promising positron emission tomography (PET) tracers designed for selective imaging of the vesicular acetylcholine transporter (VAChT), which is a direct measure of presynaptic cholinergic terminal density (Bohnen et al., 2018, Petrou et al., 2014). Albin et al. (2018) recently described detailed FEOBV binding in 29 normal adults, which was consistent with findings from the prior animal and post-mortem human studies (Parent et al., 2013a, Parent et al., 2013b). Previous FEOBV PET studies in AD patients showed reduced uptake spanning lateral fronto-parietal and temporal cortical areas as well as positive correlations between the average cortical FEOBV uptake and global cognitive measures (Aghourian et al., 2017, Schmitz et al., 2018). More extensive cholinergic denervation was demonstrated in dementia with Lewy bodies using FEOBV PET that covered cortical and subcortical areas involved in key neural hubs (Kanel et al., 2020, Nejad-Davarani et al., 2019). The cortical cholinergic denervation observed in FEOBV images was also found to associate with reduced performance in multiple cognitive domains for cognitively unimpaired (CU) patients with Parkinson’s disease (van der Zee et al., 2021). However, the relationship between cholinergic FEOBV binding and the clinical/pathological features of dementia have not been fully explored.

To our knowledge, there is no study that assessed the cortical cholinergic denervation *in vivo* at the prodromal stage of dementia, in order to explore the relative influence of cholinergic BF dysfunction and region-specific cholinergic denervation on cognitive performance at an early stage. In this study, we aimed to.

(i) evaluate the use of FEOBV PET for direct imaging of the cholinergic terminal integrity in the cortex with a sample of CU individuals and participants with MCI and.

(ii) examine the spatial topography of cholinergic denervation measured by FEOBV PET in correlation with cognitive impairment.

## 2 Material and methods

### 2.1 Study design

We conducted a cross-sectional, groupwise study designed to investigate the utility of FEOBV PET imaging for direct and quantitative assessment of cholinergic terminal integrity in a cohort of participants with milder disease in the spectrum of AD. This allowed treatment-free cases to be captured in order to assess the feasibility of the study design. Male and female participants aged 55 years or older from various cultural and linguistic backgrounds, who were fluent in both written and spoken English, were invited to participate in this study. Ethics approval was granted for the study by The Prince Charles Hospital (TPCH) Human Research Ethics Committee.

Participants were recruited from a single site, metropolitan hospital memory clinic at TPCH. All cognitively impaired participants met the Petersen criteria for MCI including memory complaint, normal activities of daily living, normal general cognitive function with Mini-Mental State Examination (MMSE) ≥ 24, abnormal memory for age and lack of dementia (Petersen et al., 1999). CU participants were primarily the spouses of patients from the memory clinic who have not been recruited into this study, in addition to other independent research volunteers. Participants were excluded if there was evidence of head trauma, a primary psychiatric diagnosis, any infectious or endocrine cause of cognitive dysfunction, a Geriatric Depression Scale score > 16/30, alcohol consumption>30 g per day in men and 20 g per day in women, or a history of habituation to drugs such as benzodiazepines or narcotics. Informed written consent was obtained from participants in both groups using separate consent forms.

All participants were reviewed by a consultant geriatrician and underwent a full physical examination, including height, weight, and vital observations, as well as blood tests. A blood sample for determination of apolipoprotein E (APOE) genotype status was collected and analysed. The same protocol was used for the CU and MCI groups, including neuropsychological assessment and brain imaging.

### 2.2 Neuropsychology assessment

Within 2 weeks of enrolment, cognitive assessments for the participants were conducted by an experienced neuropsychologist. The assessment scales included in the neuropsychological battery of this study are listed in Table 1.

**Table 1:** Neuropsychology assessment scales used in this study.

	Assessment Scales
1	Mini-Mental State Examination (Folstein et al., 1975)
2	General orientation
3	Advanced Clinical Solutions Test of Premorbid Functioning (Pearson, 2009), a revision of the Wechsler Test of Adult Reading (Wechsler, 2001)
4	Informant Questionnaire on Cognitive Decline in the Elderly (IQCODE) – Short Form (Jorm, 2004) - completed by a knowledgeable informant
5	Boston Naming Test – Short Form (Saxton et al., 2000)
6	Wechsler Memory Scale – Fourth Edition (WMS-IV; Visual Reproductions I and II + Recognition, and Symbol Span subtests) (Wechsler, 2009)
7	Rey Auditory Verbal Learning Test I and II (Ivnik et al., 1990)
8	Wechsler Adult Intelligence Scale – Fourth Edition (WAIS-IV; Digit Span subtests and Block Design subtest) (Wechsler, 2008)
9	Controlled Oral Word Association Test (COWAT; FAS) and Semantic Fluency Test (Animals) (Tombaugh et al., 1999)
10	Victoria Stroop Test (Troyer et al., 2006)
11	Austin Maze Test (Version 2.6) - Application for iPad (Darby and Walsh, 2005)
12	Trail-Making Tests A and B (Reitan, 1958)
13	Geriatric Anxiety Inventory (GAI) – 20-item research version adapted from Pachana et al. (2007). In comparison to the dichotomous yes/no format of the original GAI, items on the research version are rated on a four-point scale (i.e., *disagree, slightly agree, moderately agree, strongly agree*) to increase the range of scores (N. A. Pachana, personal communication, 15 January 2016).
14	Geriatric Depression Scale (GDS) (Yesavage et al., 1982) – 15-item research version. In keeping with the format of the GAI, items on the GDS were also modified to a four-point scale (N. A. Pachana, personal communication, 15 January 2016).

All the raw scores of cognitive measures were converted to standardized z-scores based on the well-established normative data. Composite scores for cognitive domains of memory, executive function, attention, and language were calculated by averaging z-scores of the tests or subtasks related to each of the cognitive domains using the guidelines from previous studies (Crane et al., 2012, Fowler et al., 2021, Gibbons et al., 2012):

• **Memory**: Rey Auditory Verbal Learning Test – short delay & long delay, Wechsler Memory Scale - Visual Reproduction I & II,
• **Executive function**: Trail-Making Test B, Controlled Oral Word Association Test, Wechsler Adult Intelligence Scale - Digit Span (Backwards),
• **Attention:** Trail-Making Test A, Victoria Stroop Test,
• **Language**: Boston Naming Test, Semantic Fluency Test (Animals).

A higher composite score reflects better cognitive task performance.

### 2.3 Brain imaging

The PET scans were performed on a Biograph mMR hybrid scanner (Siemens Healthineers, Erlangen, Germany).

For the [^18^F]-florbetaben (FBB) PET studies, a radiotracer for assessment of Aβ deposition (Rowe et al., 2017), a 20-minute scan was acquired starting at 90 min post injection of 300 ± 10% MBq FBB. For the concurrent MRI, ultrashort echo-time (UTE) images were first acquired and UTE-based segmentation was visually checked for attenuation correction. A T1-weighted structural image was also acquired using the 3D magnetization-prepared rapid gradient echo (MPRAGE) sequence.

For the [^18^F]-FEOBV PET studies, a radiotracer for direct imaging of cholinergic terminal integrity, 30-minute dynamic scans were acquired at 90 min and 180 min post-bolus injection of 240 ± 10% MBq FEOBV in order to estimate the tissue time activity curves (Supplementary Fig. 1). At the 180-minute scanning session, a static FEOBV image was also calculated by averaging the co-registered image frames from a subset of the dynamic imaging data within a 20-minute time window. Visual inspection of all PET scans was performed prior to image processing. Concurrent T1-weighted 3D MPRAGE images were acquired with the related acquisition parameters being echo time/repetition time = 2.26 *ms*/2.3 *s*, inversion time = 0.9 *s*, flip angle 8°, 1 *mm* isotropic resolution, and matrix 256x240x192.

### 2.4 Image processing

#### 2.4.1 Amyloid-β PET assessment

Aβ burden was automatically quantified in Centiloid from FBB PET scans using CapAIBL (Bourgeat et al., 2015), a PET-only approach that allows Aβ quantification with no bias from MRI features. Aβ burden was estimated in terms of the Centiloid values using the standard Statistical Parametric Mapping (SPM) centiloid cortical mask (Bourgeat et al., 2018, Klunk et al., 2015) and non-negative matrix factorization-based quantification (Bourgeat et al., 2021). A Centiloid threshold of 20 was used to determine abnormal levels of Aβ deposition (Aβ+ ), as validated using autopsy data (Doré et al., 2019).

#### 2.4.2 MR image processing

The 3D MPRAGE images acquired from the FEOBV PET session were used for image processing. The images were first spatially normalised in the Montreal Neurological Institute (MNI) space and segmented into grey matter (GM), white matter (WM) and cerebrospinal fluid tissues using an in-house implementation of the expectation maximization algorithm (Leemput et al., 1999). The brain parcellation was performed based on the Automated Anatomical Labeling (AAL) parcellation atlas using Learning Embeddings for Atlas Propagation (Wolz et al., 2010), which provided the regions of interest (ROI) in the MNI space for regional FEOBV quantification. The hippocampus was segmented using a multi-atlas approach based on the Harmonized Hippocampus Protocol (Boccardi et al., 2015).

BF volumes were quantified from the MPRAGE images using an SPM-based group-wise segmentation pipeline and a published cytoarchitectonic map of the BF cholinergic nuclei (Kerbler et al., 2015, Zaborszky et al., 2008). The BF mask comprises four groups of cholinergic neurons (Ch1-4): the medial septal nucleus (Ch1), the nucleus of the vertical and horizontal limb of the diagonal band of Broca (Ch2 and Ch3) and the NBM (Ch4). The sum of these volumes per participant – the total BF volume – was the measure used for our analyses.

#### 2.4.3 FEOBV PET processing

Static FEOBV PET images were co-registered to the MPRAGE images, and spatially normalized to MNI space, where the corresponding brain tissue segmentation and parcellation masks were applied to define the ROIs for regional FEOBV quantification. The related WM segmentation was used to create a supratentorial WM mask using morphological operations, which was then used as the reference region to normalize the FEOBV PET images (Nejad-Davarani et al., 2019). Supplementary Fig. 1 demonstrates the average time-activity curves of the supratentorial WM region within the CU and MCI groups. Regional FEOBV standardized uptake value ratios (SUVRs) were quantified as the 15% trimmed mean values for selected ROIs (including the cortical GM, frontal lobe, temporal lobe, parietal lobe, occipital lobe, hippocampus, amygdala, and thalamus) defined in the AAL parcellation mask.

### 2.5 Data analysis

One-way analysis of variance (ANOVA) or the Kruskal-Wallis rank sum test was used for group comparisons for continuous data including age, years of education, MMSE, neuroimaging measures and cognitive scores. Categorical data were compared using Fisher’s exact test for the small sample size. Volumetric measures (i.e., hippocampal and BF volumes) were corrected for the total intracranial volume (TIV) prior to the data analysis. Age, sex and education were used as common covariates. APOE *ε*4 carriage was not considered as a covariate as no significant group difference was observed (Table 2).

**Table 2:** Demographic and neuropsychological characteristics and multimodal imaging measures of the cohort.

	**CU**	**MCI**	**p-value**
No. of Participants	10	8	*–*
Age	78.4 ± 3.9	73.1 ± 7.8	0.110^1^
Female, n (%)	6 (60%)	4 (50%)	1.0^3^
Education, y	12.9 ± 3.4	11.3 ± 4.1	0.263^1^
IQCODE	47.1 ± 8.3	64.5 ± 3.8	< 0.001***^1^
MMSE	29.50 ± 0.71	27.62 ± 1.51	0.009**^1^
APOE e4, n (%)	3 (30%)	2 (25%)	1.0^3^
Memory CS	0.99 ± 0.81	−1.15 ± 1.33	0.002**^2^
Executive Function CS	0.77 ± 0.75	−0.61 ± 1.20	0.026*^2^
Attention CS	0.70 ± 0.70	−0.34 ± 0.69	0.018*^2^
Language CS	0.69 ± 0.55	−1.00 ± 1.00	< 0.001***^2^
Centiloid	12.2 ± 24.0	17.8 ± 49.5	0.910^2^
Aβ+, n (%)	4 (40.0%)	2 (25%)	0.367^3^
GM (*ml*)	566.8 ± 21.5	549.6 ± 30.2	0.014*^2^
Hippocampus (*ml*)	5.69 ± 0.43	5.02 ± 0.77	0.004**^2^
Basal Forebrain (*ml*)	0.49 ± 0.05	0.43 ± 0.08	0.032*^2^

CS = Composite Score. All the variables are presented as mean ± standard deviation. The p-values are computed using ^1^Kruskal-Wallis rank sum test, ^2^One-way ANOVA test correcting for age, sex, and education or ^3^ Fisher’s exact test of independence.

Similarly, the global and regional cholinergic terminal integrity measured as FEOBV SUVR was compared between the CU and MCI groups using one-way ANOVA analysis, adjusting for age, sex and education. The effect size of group differences in regional FEOBV SUVRs was measured by the Cohen’s *d*, where an effect size of 0.2 is small, 0.5 is medium, 0.8 is large (Cohen, 1988). The associations of the global FEOBV SUVR and other neuroimaging/cognitive measures were examined in the full cohort using the linear regression model, adjusting for age, sex and education, and partial correlation coefficient *r* was used to measure the strength of the association. For all analyses, a *p* value of <0.05 was used to indicate the statistical significance. Multiple comparisons were corrected using the Benjamini-Hochberg method when needed.

An exploratory whole brain voxel-wise analysis was conducted to investigate the anatomical specificity of FEOBV retention that correlated with the group status of clinical diagnosis as well as cognitive performance across different domains. Voxel-based morphometry (VBM) was first performed on the structural images using the standard FSL-VBM pipeline, which created a study-specific GM template using 8 randomly chosen CU controls and all 8 participants with MCI. Non-linear deformations were estimated from the native GM images to this template space. The FEOBV SUVR images were first registered onto their corresponding structural images and then normalized to this template space using the related non-linear deformations. Similarly, the native GM images were normalized to the same template space and modulated to correct for volume changes due to spatial transformation. A 4-mm Gaussian kernel was used to smooth the modulated GM images and no smoothing was applied to the FEOBV images due to strong correlation between the adjacent voxels in the PET images. Voxel-wise analyses of FEOBV and GM images were performed using nonparametric permutation tests (5,000 permutations) within a common GM ROI mask, which was defined from the average GM image using a threshold of 0.35 in order to ensure that only the effective GM voxels were tested. All voxel-wise analyses were corrected for age, sex and TIV as well as for multiple comparisons using the threshold free cluster enhancement (TFCE) method. In addition, small clusters (TFCE-corrected *p* < 0.05) with fewer than 50 voxels (i.e., 0.4 *ml*) were excluded. The post hoc analysis was performed on regional FEOBV SUVR values for the identified AAL ROIs using the linear regression model, adjusting for age, sex and education.

## 3 Results

### 3.1 Recruitment overview and participant characteristics

Twenty-three participants were recruited for the study, of whom 18 participants (8 MCI and 10 CU) completed all the neuropsychological assessment and brain imaging sessions.

Demographic and neuropsychological characteristics for this study cohort (N = 18) are summarised in Table 2. Participants in the CU group were slightly older than the participants with MCI but the difference was not significant (*p* = 0.110). Significant evidence of cognitive decline in the MCI group was observed in all four cognitive domains (all *p* < 0.05). For Aβ burden, there were 4 Aβ + participants in the CU group (40%) and 2 in the MCI group (25%). No group difference of Aβ deposition in terms of the binary status or Centiloid value was observed. Significantly smaller GM, hippocampal and BF volumes were observed in the MCI participants (all *p* < 0.05, Table 2).

### 3.2 Associations with FEOBV cholinergic biomarkers

**Fig. 1:**
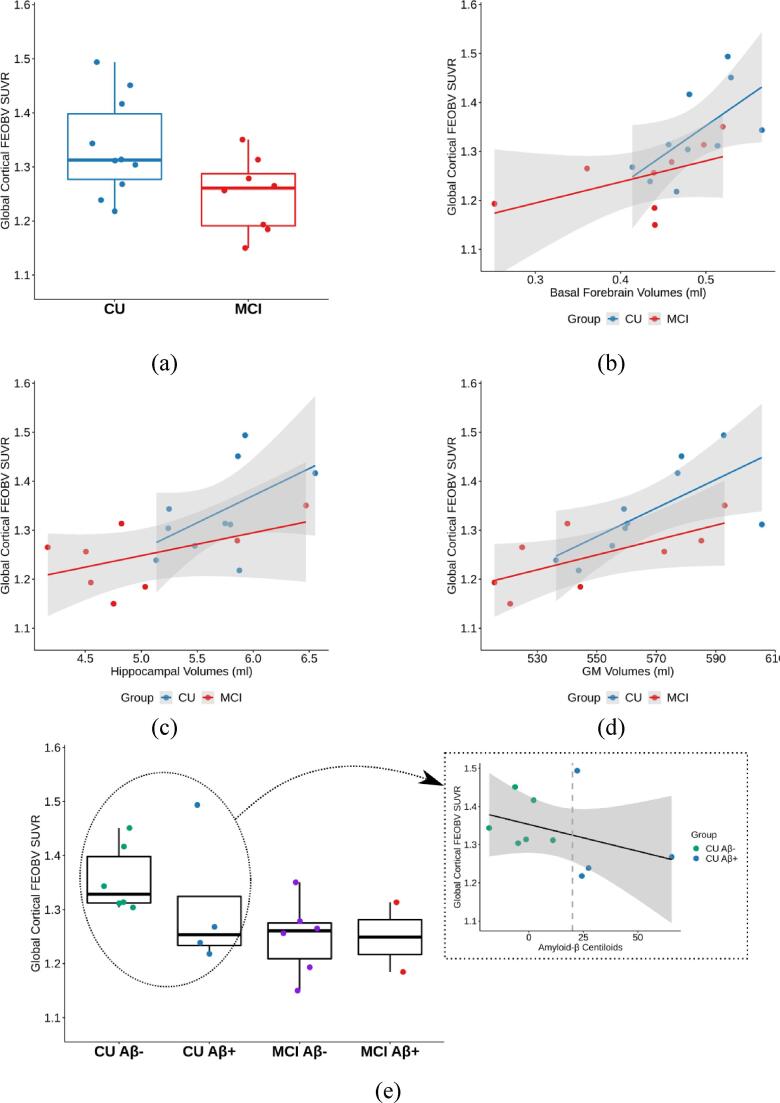
Correlation plots between global cortical FEOBV SUVR and (a) clinical diagnosis status, (b) BF volumes, (c) hippocampal volumes, (d) GM volumes, and (e) Aβ status/deposition.

**Fig. 2:**
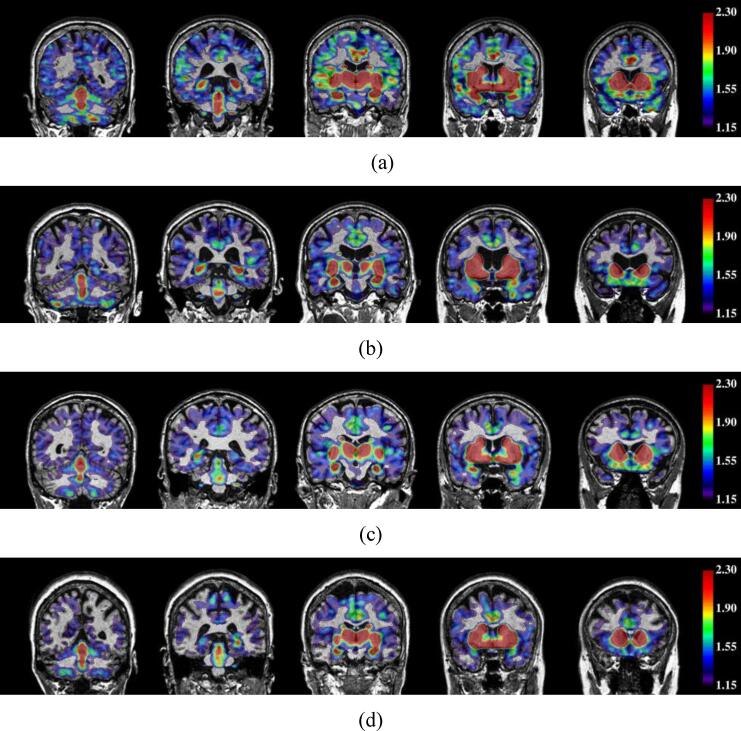
Example FEOBV SUVR images overlayed on structural MR images with global cortical FEOBV SUVR = 1.417, 1.268, 1.256 and 1.185 respectively for (a) Aβ- CU, (b) Aβ+ CU, (c) Aβ- MCI and (d) Aβ+ MCI participants. Regions without the overlaid colour indicate the FEOBV SUVR values to be < 1.15.

The global and regional SUVRs per cortical lobe for FEOBV PET are summarized in Table 3. Participants with MCI had a significantly lower FEOBV SUVR (1.25 ± 0.07) in the cortex than CU participants (1.34 ± 0.09, Fig. 1a), with Cohen’s *d* = 1.061 (large effect size) and *p* = 0.042 after adjustment for age, sex and education. Fig. 2 demonstrates the cholinergic binding patterns from FEOBV PET images for examples with different Aβ status from the CU and MCI groups, respectively. Reduced FEOBV retention in the MCI group was also observed in several cortical lobes (frontal, parietal, occipital) and the amygdala with all Cohen’s *d* > 0.8 (large effect size), where a significant group-wise difference was observed for the parietal and occipital cortices (*p* = 0.030 and 0.027, respectively). However, the observed differences became non-significant after correcting for multiple comparisons.

**Table 3:** Regional FEOBV SUVR values (Mean ± SD) between the CU and MCI groups.

Region	CU (N = 10)	MCI (N = 8)	F-value	*p*-value^1^	Cohen’s *d*
Global Cortex	1.34 ± 0.09	1.25 ± 0.07	5.062	0.042	1.061
Frontal Cortex	1.38 ± 0.08	1.30 ± 0.05	3.889	0.070	1.181
Temporal Cortex	1.31 ± 0.11	1.25 ± 0.11	1.624	0.225	0.612
Parietal Cortex	1.28 ± 0.09	1.18 ± 0.08	5.936	0.030	1.185
Occipital Cortex	1.19 ± 0.09	1.11 ± 0.08	6.261	0.027	0.956
Hippocampus	1.97 ± 0.21	1.85 ± 0.21	2.435	0.143	0.562
Amygdala	2.56 ± 0.26	2.33 ± 0.21	4.331	0.058	0.942
Thalamus	2.08 ± 0.20	2.07 ± 0.21	0.994	0.337	0.076

1 The *p*-values were computed using one-way ANOVA test, adjusting for age, sex and education, and have not been adjusted for multiple comparisons. No p-values were<0.05 after correcting for multiple comparisons.

When combining all participant data, the global cortical FEOBV SUVR significantly correlated with BF volume (*r* = 0.630, *p* = 0.012 in one-way ANOVA analysis with adjustment of age, sex and education, Fig. 1b), hippocampal volume (*r* = 0.587, *p* = 0.022, Fig. 1c) as well as GM volume (*r* = 0.763, *p* < 0.001, Fig. 1d). There was no significant association with Aβ status observed in the entire cohort or within each group, although a trend of negative correlation (not statistically significant) between the global FEOBV SUVR and Centiloid values was noted within the CU group (Fig. 1e). When entering the group status and its interaction with volumetric measures in the model, the global cortical FEOBV SUVR remained significantly correlated with the BF and GM volume (*r* = 0.529, *p* = 0.020 and *r* = 0.666, *p* = 0.017, respectively) but not with hippocampal volume (*r* = 0.369, *p* = 0.168).

When associations with cognitive performance profiles were assessed in the full cohort, positive correlations were found between the global cortical FEOBV SUVR and cognitive composite scores for executive function (*r* = 0.708, *p* = 0.004), attention (*r* = 0.605, *p* = 0.017) and language (*r* = 0.542, *p* = 0.037) after adjusting for age, sex and education. The observed correlations remained significant after correcting for multiple testing. For the performance in memory, the correlation with the global FEOBV SUVR was close to the level of significance (*r* = 0.502, *p* = 0.057).

### 3.3 Voxel-Wise analysis of FEOBV PET across cognitive domains

**Fig. 3:**
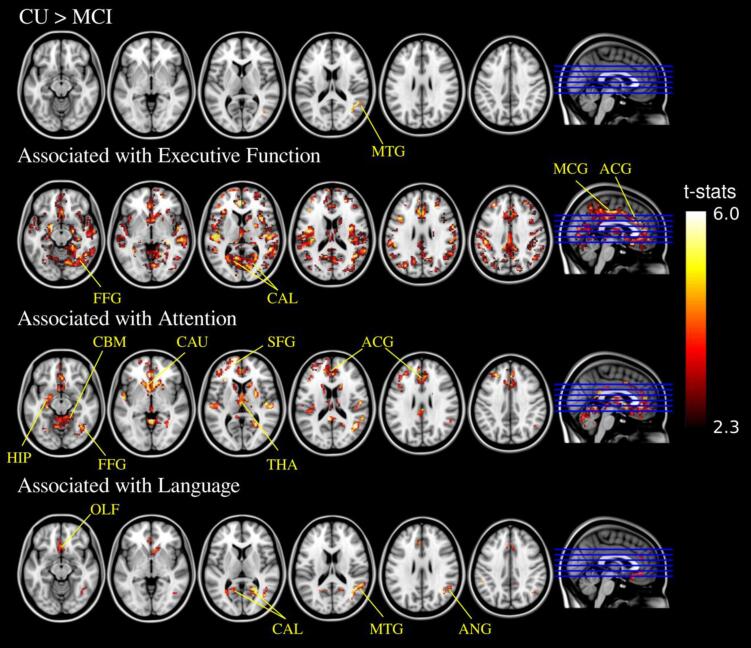
Voxel-wise analysis (TFCE-corrected *p* < 0.05) of the correlation between FEOBV SUVR and (top – bottom) group status of clinical diagnosis, executive function composite scores, attention composite scores, language composite scores. MTG = middle temporal gyrus, FFG = fusiform gyrus, CAL = calcarine cortex, MCG = median cingulate gyrus, ACG = anterior cingulate gyrus, CBM = cerebellum, CAU = caudate, THA = thalamus, SFG = superior frontal gyrus, OLF = olfactory cortex, ANG = angular gyrus.

**Fig. 4:**
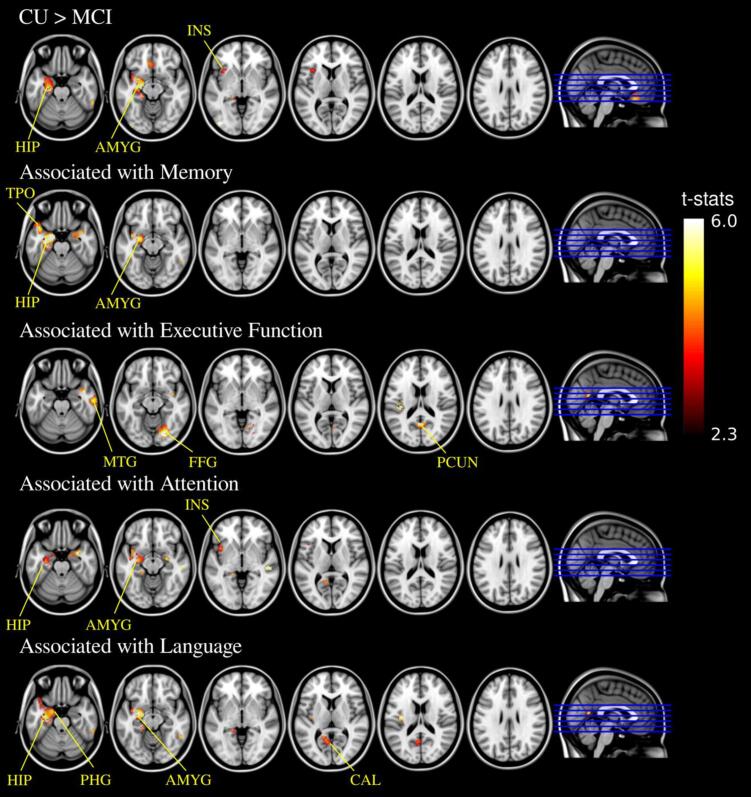
Voxel-wise analysis (TFCE-corrected *p* < 0.05) of the correlation between GM density and (top – bottom) group status of clinical diagnosis, memory composite scores, executive function composite scores, attention composite scores, language composite scores. HIP = hippocampus, AMYG = amygdala, INS = insula, TPO = temporal pole, MTG = middle temporal gyrus, FFG = fusiform gyrus, PCUN = precuneus, PHG = parahippocampal gyrus, CAL = calcarine cortex.

Significant FEOBV SUVR reductions (TFCE-corrected *p* < 0.05) among the participants with MCI comparing to the CU individuals were found in a small focal cluster (1.2 *ml*) near the middle temporal gyrus in the right hemisphere (*t*-value = 4.583 ± 0.597, Fig. 3). In contrast, significant reduction in GM density was identified in several cortical and subcortical structures in the left hemisphere (*t*-value = 4.296 ± 0.730), which mainly included the hippocampus, amygdala, parahippocampal gyrus, and insular cortex (Fig. 4).

Most interestingly, regional positive correlations (TFCE-corrected *p* < 0.05) were found between FEOBV retention and cognitive scores across different cognitive domains, although these did not include memory (Fig. 3). No significant negative correlations with FEOBV retention were observed for any cognitive domain. This indicates that lower FEOBV retention is associated with reduced performance in several cognitive domains. For executive functioning, positive correlations with FEOBV retention were widespread and covered both cortical and subcortical regions. For the domain of attention, clusters with positive correlations were located mainly in the anterior cingulate gyrus, left superior frontal gyrus, caudate nucleus, thalamus, left hippocampus and cerebellum. For the domain of language, clusters with positive correlations were found mainly in the olfactory cortex, calcarine cortex and right middle temporal gyrus. The average *t*-values in these correlation clusters were 3.167 (*SD* = 0.800), 3.588 (*SD* = 0.788), and 3.923 (*SD* = 0.698) for executive function, attention, and language, respectively.

Common clusters were identified that showed positive correlations with the FEOBV retention for all three cognitive domains of executive function, attention and language, which had a total size of 8.9 *ml* (1,111 voxels) covering the anterior cingulate gyrus, olfactory cortex, calcarine cortex, fusiform gyrus, angular gyrus, precuneus cortex, middle temporal gyrus and caudate nucleus. These overlapping topographic profiles were mapped to the AAL parcellation regions, with Table 4 summarizing linear regression models for the identified AAL ROIs describing the association between the regional FEOBV SUVR and domain-specific cognitive performance. Following the post-hoc analysis, positive correlations were observed between regional FEOBV SUVR and memory performance in some regions from the overlapping topographic profiles, such as the middle cingulate and paracingulate gyri, calcarine fissure, lingual gyrus and precuneus cortex, before correcting for multiple testing (Table 4).

**Table 4:** Linear regression models with the partial correlation coefficients and *p*-values showing the correlations between regional FEOBV SUVR and domain-specific scores across all participants.

AAL Regions	Memory	Executive Function	Attention	Language
Olfactory cortex	0.458 *p* = 0.100	0.575 *p* = 0.032*	0.626 *p* = 0.017*	0.457 *p* = 0.101
Anterior cingulate and paracingulate gyri	0.366 *p* = 0.199	0.593 *p* = 0.026*	0.673 *p* = 0.009*	0.450 *p* = 0.107
Middle cingulate and paracingulate gyri	0.627 *p* = 0.017	0.755 *p* = 0.002*	0.684 *p* = 0.008*	0.649 *p* = 0.012*
Calcarine fissure	0.641 *p* = 0.014	0.719 *p* = 0.004*	0.671 *p* = 0.009*	0.749 *p* = 0.003*
Lingual gyrus	0.547 *p* = 0.043	0.758 *p* = 0.002*	0.726 *p* = 0.004*	0.659 *p* = 0.011*
Fusiform gyrus	0.498 *p* = 0.071	0.624 *p* = 0.017*	0.628 *p* = 0.017*	0.505 *p* = 0.066
Angular gyrus	0.496 *p* = 0.072	0.724 *p* = 0.004*	0.607 *p* = 0.022*	0.570 *p* = 0.034
Precuneus	0.562 *p* = 0.037	0.742 *p* = 0.003*	0.685 *p* = 0.007*	0.663 *p* = 0.010*
Middle temporal gyrus	0.369 *p* = 0.195	0.636 *p* = 0.015*	0.571 *p* = 0.034*	0.361 *p* = 0.206
Caudate nucleus	0.329 *p* = 0.251	0.514 *p* = 0.061	0.739 *p* = 0.003*	0.540 *p* = 0.047

All the models were corrected for age, sex and education. The reported *p*-values have not been adjusted for multiple comparisons. * indicates the significance level *p* < 0.05 after correcting for multiple comparisons.

The VBM analysis of GM density, revealed overlapping clusters with a total size of 4.4 *ml* (555 voxels) near the hippocampus, amygdala and parahippocampal gyrus in the left hemisphere that significantly correlated with memory, attention, and language scores (Fig. 4). For the domain of executive function, positive correlation clusters were located in the lingual gyrus, fusiform gyrus, and middle temporal gyrus in the right hemisphere as well as the precuneus cortex. These distinctive patterns in FEOBV SUVR and GM density in association with domain-specific cognitive scores indicate that lower cholinergic terminal integrity associated with cognitive impairment was not accompanied by measurable local cortical atrophy (Fernández-Cabello et al., 2020).

## 4 Discussion

The findings from this study provide support for the utility of FEOBV PET for quantitative detection of cortical cholinergic degeneration in MCI participants. Reduced cholinergic innervation quantified from FEOBV PET images was observed globally and in parietal and occipital cortices among the participants with MCI (all *p* < 0.05 before adjusting for multiple testing). These results are consistent with previous reports that patients with cognitive impairment had reduced acetylcholinesterase (AChE) activity in the lateral temporal, parietal, and occipital cortices and, to a lesser degree, in the frontal cortex (Haense et al., 2012, Richter et al., 2019). Secondly, the global FEOBV retention was found to positively correlate with executive function, attention, and language. Similar positive correlations have been reported between the average cortical FEOBV retention with MMSE and related global cognitive scales (Aghourian et al., 2017). These associations with cognitive functioning are also in line with previous work based on MRI-based BF atrophy (Grothe et al., 2016).

A strong positive correlation (*r* = 0.630, *p* = 0.012) was found for the full cohort between the global cortical FEOBV SUVR and BF volumes, which may indicate a link between the loss of BF cholinergic neurons (resulting in BF atrophy) and reduced cholinergic innervation throughout the cortex (Kilimann et al., 2017, Schmitz et al., 2018). This correlation seems to also involve the effect of the global brain atrophy as similar positive correlations were observed between FEOBV SUVR and hippocampal/GM atrophy (Fig. 1c and 1d). Studies on a larger cohort of participants at preclinical stages of dementia (before significant widespread brain atrophy occurs) are needed to confirm the association between the BF atrophy and reduced cortical cholinergic innervation. Future analyses that investigate sub-regional BF atrophy in association with FEOBV binding in the cortical and subcortical GM (e.g., hippocampus) would also be useful to directly demonstrate the corticotopic organization of BF cholinergic system projections in relation to cognitive domains.

In our cohort of participants diagnosed with MCI, a low prevalence of Aβ+ (i.e., 25%) was observed comparing to 69% of MCI reported in literature (Villemagne et al., 2011). This could be attributed to the small sample size (N = 8) with a low prevalence of APOE ε4 carriage (i.e., 25%) as well as a relatively high average MMSE score of 27.6 in this MCI group. Additionally, while the Aβ- MCI participants might progress to dementia, it is unlikely they will progress to AD given that they do not have AD pathology, therefore they cannot be classified as prodromal AD. It was perhaps unsurprising that we did not observe a significant association between FEOBV retention and Aβ deposition, particularly with our small sample size. However, the observed trend in the CU group as shown in Fig. 1e may indicate a potential correlation of cortical FEOBV binding and Aβ deposition at the preclinical stage of AD, which needs to be further confirmed with a much larger cohort across the AD spectrum.

Our findings were further expanded by using whole-brain voxel-wise analysis of FEOBV images. Topographic reductions in FEOBV binding correlated with domain-specific cognitive impairment, which is in line with the current literature reporting strong correlations between regional AChE activity and various neuropsychological measures for different cognitive functions (Haense et al., 2012). Importantly, while BF volume loss has been reported to precede cortical atrophy, particularly in those areas of high Aβ deposition (Fernández-Cabello et al., 2020), voxel-wise analyses of GM density only revealed several focal reductions in the left hippocampus and amygdala and right temporal cortex that were associated with decreased cognitive functioning. This suggests that the use of FEOBV to assess cholinergic terminal integrity may provide an early indicator of loss of cortical function. In addition, FEOBV PET, with the provision of valuable information to detect the regional pattern of the cholinergic terminal integrity, would potentially facilitate the identification of distinct vulnerable areas in association with the diverse cognitive profiles seen in dementia at the prodromal stage.

From a neurocognitive perspective, the topographic profiles of domain-specific neuropsychological correlations could reflect, to some extent, the involvement of the key functional networks in cognitive functioning, and that reduced cholinergic terminal integrity within these networks is associated with poorer performance on selected cognitive tasks requiring domain-specific skills. More specifically, in terms of executive functioning, correlation clusters identified in the prefrontal cortex, middle cingulate gyrus, and inferior parietal gyrus indicate cholinergic denervation patterns within the fronto-parietal network, while clusters in anterior cingulate gyrus, insula, and thalamus reflect cholinergic denervation patterns within the cingulo-opercular network. These patterns are consistent with the well-established dual-network model of executive function, supporting complex cognitive processes such as working memory, sustained attention, and executive control (Dosenbach et al., 2008). Moreover, correlation clusters in the calcarine cortex and lingual gyrus were identified for the domains of both executive function and language, which could reflect the involvement of the visual network in measures such as Trial-Making Test B (for assessing visual attention and mental flexibility) and the Boston Naming Test (for assessing visual confrontation naming).

Additionally, clusters of FEOBV binding in the cerebellum were identified as being associated with performance on assessments of executive functioning and attention (Fig. 3). Although the cerebellum principally contributes to motor control and coordination, increasing evidence has shown that there is cerebellar involvement in multiple neuropsychological domains including executive function and attention (Bellebaum and Daum, 2007, O'Halloran et al., 2012). Indeed, recent studies using functional MRI have demonstrated activation within the cerebellum during performance of specific cognitive tasks such as trail-making and Stroop tests (Rocca et al., 2009, Talwar et al., 2020).

In contrast, no identified cluster of FEOBV binding was significantly associated with memory performance on the assessments administered, potentially due to the small sample size. Interestingly, a post-hoc analysis revealed that several brain regions from the overlapping topographic profiles of neuropsychological correlates did show positive relationship between regional FEOBV SUVR and memory performance, although none of these correlations reached statistical significance after correcting for multiple comparisons (Table 4). The findings suggest that these regions may be involved in key functional networks serving multiple cognitive domains, including memory, and warrant further investigation in larger cohorts.

The identification of patients with MCI at risk of conversion is still one of the challenges in clinical practice as 20–40% of them may not progress to dementia and are affected by a variety of conditions rather than Alzheimer’s pathology (Barnes et al., 2006, Mitchell and Shiri-Feshki, 2009). Future follow-up studies of our MCI group will examine whether the patterns of FEOBV retention that correlate with cognitive profiles in different domains could be used for predicting the conversion risk for dementia. Meanwhile, future studies in larger cohorts will be needed to confirm these initial observations and to extend research based on multiple image modalities (e.g., MRI, PET). This would provide additional information on structural and functional cholinergic integrity to facilitate the development of novel imaging biomarkers for quantitative assessment of cortical cholinergic innervation in ageing and dementia.

In conclusion, we have demonstrated that the topographic profile of FEOBV retention correlated with reduced cognitive performance in different domains, suggesting that lower cortical cholinergic terminal integrity is an important pathological feature of cognitive decline. Our study therefore provides promising proof of concept that FEOBV PET could be a direct and quantitative tool to assess region-specific cholinergic denervation in the cortex, which could open the door to prognostication of therapy response and therefore personalisation of treatment (e.g., with cholinesterase inhibitors). Additionally, FEOBV PET could serve as a promising imaging biomarker for cholinergic terminal integrity and may present a valuable research tool for future trials.

### CRediT authorship contribution statement

**Ying Xia:** Software, Formal analysis, Investigation, Writing – original draft, Writing – review & editing. **Eamonn Eeles:** Conceptualization, Methodology, Investigation, Resources, Funding acquisition, Writing – review & editing. **Jurgen Fripp:** Conceptualization, Methodology, Supervision, Funding acquisition, Writing – review & editing. **Donna Pinsker:** Methodology, Investigation, Data curation, Writing – review & editing. **Paul Thomas:** Resources, Writing – review & editing. **Melissa Latter:** Resources, Writing – review & editing. **Vincent Doré:** Resources, Writing – review & editing. **Amir Fazlollahi:** Investigation, Writing – review & editing. **Pierrick Bourgeat:** Software, Resources, Writing – review & editing. **Victor L. Villemagne:** Conceptualization, Writing – review & editing. **Elizabeth J. Coulson:** Conceptualization, Methodology, Supervision, Funding acquisition, Writing – review & editing. **Stephen Rose:** Conceptualization, Methodology, Supervision, Funding acquisition, Writing – review & editing.

## Declaration of Competing Interest

The authors declare that they have no known competing financial interests or personal relationships that could have appeared to influence the work reported in this paper.

## References

[b0005] Aghourian M., Legault-Denis C., Soucy J.P., Rosa-Neto P., Gauthier S., Kostikov A., Gravel P., Bédard M.A. (2017). Quantification of brain cholinergic denervation in Alzheimer’s disease using PET imaging with [18F]-FEOBV. Mol. Psychiatry.

[b0010] Albin R.L., Bohnen N.I., Muller M.L.T.M., Dauer W.T., Sarter M., Frey K.A., Koeppe R.A. (2018). Regional vesicular acetylcholine transporter distribution in human brain: A [18F]fluoroethoxybenzovesamicol positron emission tomography study. J. Comparative Neurol..

[b0015] Ballinger E.C., Ananth M., Talmage D.A., Role L.W. (2016). Basal forebrain cholinergic circuits and signaling in cognition and cognitive decline. Neuron.

[b0020] Barnes D.E., Alexopoulos G.S., Lopez O.L., Williamson J.D., Yaffe K. (2006). Depressive symptoms, vascular disease, and mild cognitive impairment: findings from the cardiovascular health study. Arch. Gen. Psychiatry.

[b0025] Bellebaum C., Daum I. (2007). Cerebellar involvement in executive control. The Cerebellum.

[b0030] Boccardi M., Bocchetta M., Morency F.C., Collins D.L., Nishikawa M., Ganzola R., Grothe M.J., Wolf D., Redolfi A., Pievani M., Antelmi L., Fellgiebel A., Matsuda H., Teipel S., Duchesne S., Jack C.R., Frisoni G.B. (2015). Training labels for hippocampal segmentation based on the EADC-ADNI harmonized hippocampal protocol. Alzheimer’s & Dementia.

[b0035] Bohnen N.I., Grothe M.J., Ray N.J., Müller M.L.T.M., Teipel S.J. (2018). Recent advances in cholinergic imaging and cognitive decline—revisiting the cholinergic hypothesis of dementia. Curr. Geriatrics Rep..

[b0040] Bourgeat P., Doré V., Doecke J., Ames D., Masters C.L., Rowe C.C., Fripp J., Villemagne V.L. (2021). Non-negative matrix factorisation improves Centiloid robustness in longitudinal studies. NeuroImage.

[b0045] Bourgeat P., Doré V., Fripp J., Ames D., Masters C.L., Salvado O., Villemagne V.L., Rowe C.C. (2018). Implementing the centiloid transformation for 11C-PiB and β-amyloid 18F-PET tracers using CapAIBL. NeuroImage.

[b0050] Bourgeat P., Villemagne V.L., Dore V., Brown B., Macaulay S.L., Martins R., Masters C.L., Ames D., Ellis K., Rowe C.C., Salvado O., Fripp J. (2015). Comparison of MR-less PiB SUVR quantification methods. Neurobiol. Aging.

[b0055] Cantero J.L., Atienza M., Lage C., Zaborszky L., Vilaplana E., Lopez-Garcia S., Pozueta A., Rodriguez-Rodriguez E., Blesa R., Alcolea D., Lleo A., Sanchez-Juan P., Fortea J. (2020). Atrophy of Basal Forebrain Initiates with Tau Pathology in Individuals at Risk for Alzheimer’s Disease. Cereb. Cortex.

[b0060] Cavedo E., Grothe M.J., Colliot O., Lista S., Chupin M., Dormont D., Houot M., Lehéricy S., Teipel S., Dubois B., Hampel H., Croisile B., Louis Tisserand G., Bonafe A., Ousset P.J., Rouaud O., Ricolfi F., Vighetto A., Pasquier F., Delmaire C., Ceccaldi M., Girard N., Duveau F., Sarazin M., Hippocampus Study G. (2017). Reduced basal forebrain atrophy progression in a randomized Donepezil trial in prodromal Alzheimer’s disease. Sci. Rep..

[b0065] Cohen J. (1988).

[b0070] Crane P.K., Carle A., Gibbons L.E., Insel P., Mackin R.S., Gross A., Jones R.N., Mukherjee S., Curtis S.M., Harvey D., Weiner M., Mungas D., for the Alzheimer’s Disease Neuroimaging, I. (2012). Development and assessment of a composite score for memory in the Alzheimer’s Disease Neuroimaging Initiative (ADNI). Brain Imaging Behav..

[b0075] Darby D., Walsh K. (2005).

[b0080] Doré V., Bullich S., Rowe C.C., Bourgeat P., Konate S., Sabri O., Stephens A.W., Barthel H., Fripp J., Masters C.L., Dinkelborg L., Salvado O., Villemagne V.L., De Santi S. (2019). Comparison of 18F-florbetaben quantification results using the standard Centiloid, MR-based, and MR-less CapAIBL® approaches: Validation against histopathology. Alzheimer's & Dementia.

[b0085] Dosenbach N.U.F., Fair D.A., Cohen A.L., Schlaggar B.L., Petersen S.E. (2008). A dual-networks architecture of top-down control. Trends Cogn. Sci..

[b0090] Fernández-Cabello S., Kronbichler M., Van Dijk K.R.A., Goodman J.A., Spreng R.N., Schmitz T.W., on behalf of the Alzheimer’s Disease Neuroimaging, I. (2020). Basal forebrain volume reliably predicts the cortical spread of Alzheimer’s degeneration. Brain.

[b0095] Folstein M.F., Folstein S.E., McHugh P.R. (1975). “Mini-mental state”. A practical method for grading the cognitive state of patients for the clinician. J. Psychiatr. Res..

[b0100] Fowler C., Rainey-Smith S.R., Bird S., Bomke J., Bourgeat P., Brown B.M., Burnham S.C., Bush A.I., Chadunow C., Collins S., Doecke J., Doré V., Ellis K.A., Evered L., Fazlollahi A., Fripp J., Gardener S.L., Gibson S., Grenfell R., Harrison E., Head R., Jin L., Kamer A., Lamb F., Lautenschlager N.T., Laws S.M., Li Q.-X., Lim L., Lim Y.Y., Louey A., Macaulay S.L., Mackintosh L., Martins R.N., Maruff P., Masters C.L., McBride S., Milicic L., Peretti M., Pertile K., Porter T., Radler M., Rembach A., Robertson J., Rodrigues M., Rowe C.C., Rumble R., Salvado O., Savage G., Silbert B., Soh M., Sohrabi H.R., Taddei K., Taddei T., Thai C., Trounson B., Tyrrell R., Vacher M., Varghese S., Villemagne V.L., Weinborn M., Woodward M., Xia Y., Ames D., the, A.i. (2021). Fifteen Years of the Australian Imaging, Biomarkers and Lifestyle (AIBL) Study: Progress and Observations from 2,359 Older Adults Spanning the Spectrum from Cognitive Normality to Alzheimer’s Disease. J. Alzheimer's Disease Reports.

[b0105] Gibbons L.E., Carle A.C., Mackin R.S., Harvey D., Mukherjee S., Insel P., Curtis S.M., Mungas D., Crane P.K., for the Alzheimer’s Disease Neuroimaging, I. (2012). A composite score for executive functioning, validated in Alzheimer’s Disease Neuroimaging Initiative (ADNI) participants with baseline mild cognitive impairment. Brain Imaging Behav..

[b0110] Grothe M., Heinsen H., Teipel S.J. (2012). Atrophy of the cholinergic basal forebrain over the adult age range and in early stages of Alzheimer's disease. Biol. Psychiatry.

[b0115] Grothe M.J., Heinsen H., Amaro E., Grinberg L.T., Teipel S.J., for the Alzheimer's Disease Neuroimaging, I. (2016). Cognitive correlates of basal forebrain atrophy and associated cortical hypometabolism in mild cognitive impairment. Cereb. Cortex.

[b0120] Grothe M.J., Kilimann I., Grinberg L., Heinsen H., Teipel S., Perneczky R. (2018). Biomarkers for Preclinical Alzheimer’s Disease.

[b0125] Grothe M.J., Schuster C., Bauer F., Heinsen H., Prudlo J., Teipel S.J. (2014). Atrophy of the cholinergic basal forebrain in dementia with Lewy bodies and Alzheimer’s disease dementia. J. Neurol..

[b0130] Haense C., Kalbe E., Herholz K., Hohmann C., Neumaier B., Krais R., Heiss W.-D. (2012). Cholinergic system function and cognition in mild cognitive impairment. Neurobiol. Aging.

[b0135] Hall A.M., Moore R.Y., Lopez O.L., Kuller L., Becker J.T. (2008). Basal forebrain atrophy is a presymptomatic marker for Alzheimer's disease. Alzheimer's & Dementia.

[b0140] Hampel H., Mesulam M.M., Cuello A.C., Farlow M.R., Giacobini E., Grossberg G.T., Khachaturian A.S., Vergallo A., Cavedo E., Snyder P.J., Khachaturian Z.S. (2018). The cholinergic system in the pathophysiology and treatment of Alzheimer’s disease. Brain.

[b0145] Ivnik R.J., Malec J.F., Tangalos E.G., Petersen R.C., Kokmen E., Kurland L.T. (1990). The Auditory-Verbal Learning Test (AVLT): Norms for ages 55 years and older. Psychol. Assessment: J. Consulting Clin. Psychol..

[b0150] Jorm A.F. (2004). The Informant Questionnaire on Cognitive Decline in the Elderly (IQCODE): a review. Int. Psychogeriatr..

[b0155] Kanel P., Müller M.L.T.M., van der Zee S., Sanchez-Catasus C.A., Koeppe R.A., Frey K.A., Bohnen N.I. (2020). Topography of cholinergic changes in dementia with Lewy bodies and key neural network hubs. J. Neuropsychiatry Clin. Neurosci..

[b0160] Kerbler G.M., Fripp J., Rowe C.C., Villemagne V.L., Salvado O., Rose S., Coulson E.J. (2015). Basal forebrain atrophy correlates with amyloid β burden in Alzheimer's disease. NeuroImage: Clin..

[b0165] Kilimann I., Hausner L., Fellgiebel A., Filippi M., Würdemann T.J., Heinsen H., Teipel S.J. (2017). Parallel atrophy of cortex and basal forebrain cholinergic system in mild cognitive impairment. Cereb. Cortex.

[b0170] Klunk W.E., Koeppe R.A., Price J.C., Benzinger T.L., Devous M.D., Jagust W.J., Johnson K.A., Mathis C.A., Minhas D., Pontecorvo M.J., Rowe C.C., Skovronsky D.M., Mintun M.A. (2015). The Centiloid Project: standardizing quantitative amyloid plaque estimation by PET. Alzheimer's & Dementia.

[b0175] Lammers F., Borchers F., Feinkohl I., Hendrikse J., Kant I.M.J., Kozma P., Pischon T., Slooter A.J.C., Spies C., van Montfort S.J.T., Zacharias N., Zaborszky L., Winterer G. (2018). Basal forebrain cholinergic system volume is associated with general cognitive ability in the elderly. Neuropsychologia.

[b0180] Leemput K.V., Maes F., Vandermeulen D., Suetens P. (1999). Automated model-based tissue classification of MR images of the brain. IEEE Trans. Med. Imaging.

[b0185] Mitchell A.J., Shiri-Feshki M. (2009). Rate of progression of mild cognitive impairment to dementia – meta-analysis of 41 robust inception cohort studies. Acta Psychiatr. Scand..

[b0190] Nejad-Davarani S., Koeppe R.A., Albin R.L., Frey K.A., Müller M.L.T.M., Bohnen N.I. (2019). Quantification of brain cholinergic denervation in dementia with Lewy bodies using PET imaging with [18F]-FEOBV. Mol. Psychiatry.

[b0195] O'Halloran C.J., Kinsella G.J., Storey E. (2012). The cerebellum and neuropsychological functioning: a critical review. J. Clin. Exp. Neuropsychol..

[b0200] Pachana N.A., Byrne G.J., Siddle H., Koloski N., Harley E., Arnold E. (2007). Development and validation of the Geriatric Anxiety Inventory. Int. Psychogeriatr..

[b0205] Parent M.J., Bedard M.-A., Aliaga A., Minuzzi L., Mechawar N., Soucy J.-P., Schirrmacher E., Kostikov A., Gauthier S.G., Rosa-Neto P. (2013). Cholinergic depletion in Alzheimer’s disease shown by [18F]FEOBV autoradiography. Int. J. Mol. Imaging.

[b0210] Parent M.J., Cyr M., Aliaga A., Kostikov A., Schirrmacher E., Soucy J.-P., Mechawar N., Rosa-Neto P., Bedard M.-A. (2013). Concordance between in vivo and postmortem measurements of cholinergic denervation in rats using PET with [18F]FEOBV and choline acetyltransferase immunochemistry. EJNMMI Res..

[b0215] Pearson, 2009. Advanced clinical solutions for WAIS-IV and WMS-IV: Administration and scoring manual. The Psychological Corporation, San Antonio, TX.

[b0220] Petersen R.C., Smith G.E., Waring S.C., Ivnik R.J., Tangalos E.G., Kokmen E. (1999). Mild cognitive impairment: clinical characterization and outcome. Arch. Neurol..

[b0225] Petrou M., Frey K.A., Kilbourn M.R., Scott P.J.H., Raffel D.M., Bohnen N.I., Müller M.L.T.M., Albin R.L., Koeppe R.A. (2014). In vivo imaging of human cholinergic nerve terminals with (–)-5-18F-fluoroethoxybenzovesamicol: biodistribution, dosimetry, and tracer kinetic analyses. J. Nucl. Med..

[b0230] Prado V.F., Janickova H., Al-Onaizi M.A., Prado M.A.M. (2017). Cholinergic circuits in cognitive flexibility. Neuroscience.

[b0235] Ramos-Rodriguez J.J., Pacheco-Herrero M., Thyssen D., Murillo-Carretero M.I., Berrocoso E., Spires-Jones T.L., Bacskai B.J., Garcia-Alloza M. (2013). Rapid β-amyloid deposition and cognitive impairment after cholinergic denervation in APP/PS1 mice. J. Neuropathol. Exp. Neurol..

[b0240] Reitan R.M. (1958). Validity of the trail making test as an indicator of organic brain damage. Percept. Mot. Skills.

[b0245] Richter N., Nellessen N., Dronse J., Dillen K., Jacobs H.I.L., Langen K.-J., Dietlein M., Kracht L., Neumaier B., Fink G.R., Kukolja J., Onur O.A. (2019). Spatial distributions of cholinergic impairment and neuronal hypometabolism differ in MCI due to AD. NeuroImage: Clinical.

[b0250] Rocca M.A., Valsasina P., Ceccarelli A., Absinta M., Ghezzi A., Riccitelli G., Pagani E., Falini A., Comi G., Scotti G., Filippi M. (2009). Structural and functional MRI correlates of Stroop control in benign MS. Hum. Brain Mapp..

[b0255] Rowe C.C., Doré V., Jones G., Baxendale D., Mulligan R.S., Bullich S., Stephens A.W., De Santi S., Masters C.L., Dinkelborg L., Villemagne V.L. (2017). 18F-Florbetaben PET beta-amyloid binding expressed in Centiloids. Eur. J. Nucl. Med. Mol. Imaging.

[b0260] Saxton J., Ratcliff G., Munro C.A., Coffey E.C., Becker J.T., Fried L., Kuller L. (2000). Normative data on the boston naming test and two equivalent 30-item short forms. Clin. Neuropsychol..

[b0265] Scheef L., Grothe M.J., Koppara A., Daamen M., Boecker H., Biersack H., Schild H.H., Wagner M., Teipel S., Jessen F. (2019). Subregional volume reduction of the cholinergic forebrain in subjective cognitive decline (SCD). NeuroImage: Clin..

[b0270] Schliebs R. (2005). Basal forebrain cholinergic dysfunction in Alzheimer’s disease – interrelationship with β-amyloid, inflammation and neurotrophin signaling. Neurochem. Res..

[b0275] Schliebs R., Arendt T. (2011). The cholinergic system in aging and neuronal degeneration. Behav. Brain Res..

[b0280] Schmitz T.W., Mur M., Aghourian M., Bedard M.-A., Spreng R.N. (2018). Longitudinal Alzheimer’s degeneration reflects the spatial topography of cholinergic basal forebrain projections. Cell Reports.

[b0285] Talwar N., Churchill N.W., Hird M.A., Tam F., Graham S.J., Schweizer T.A. (2020). Functional magnetic resonance imaging of the trail-making test in older adults. PLoS ONE.

[b0290] Teipel S., Heinsen H., Amaro E., Grinberg L.T., Krause B., Grothe M. (2014). Cholinergic basal forebrain atrophy predicts amyloid burden in Alzheimer's disease. Neurobiol. Aging.

[b0295] Teipel S.J., Flatz W.H., Heinsen H., Bokde A.L.W., Schoenberg S.O., Stöckel S., Dietrich O., Reiser M.F., Möller H.-J., Hampel H. (2005). Measurement of basal forebrain atrophy in Alzheimer's disease using MRI. Brain.

[b0300] Tombaugh T.N., Kozak J., Rees L. (1999). Normative data stratified by age and education for two measures of verbal fluency: FAS and animal naming. Arch. Clin. Neuropsychol..

[b0305] Troyer A.K., Leach L., Strauss E. (2006). Aging and response inhibition: normative data for the victoria stroop test. Aging Neuropsychol. Cogn..

[b0310] van der Zee S., Müller M.L.T.M., Kanel P., van Laar T., Bohnen N.I. (2021). Cholinergic denervation patterns across cognitive domains in Parkinson's disease. Mov. Disord..

[b0315] Villemagne V.L., Pike K.E., Chételat G., Ellis K.A., Mulligan R.S., Bourgeat P., Ackermann U., Jones G., Szoeke C., Salvado O., Martins R., O'Keefe G., Mathis C.A., Klunk W.E., Ames D., Masters C.L., Rowe C.C. (2011). Longitudinal assessment of Aβ and cognition in aging and Alzheimer disease. Ann. Neurol..

[b0320] Wechsler D. (2001).

[b0325] Wechsler, D., 2008. Wechsler Adult Intelligence Scale | Fourth Edition (WAIS-IV): Administration and scoring manual. The Psychological Corporation.

[b0330] Wechsler, D., 2009. Wechsler Memory Scale | Fourth Ediction (WMS-IV): Technical and interpretive manual. Pearson, San Antonio, TX.

[b0335] Wolz R., Aljabar P., Hajnal J.V., Hammers A., Rueckert D. (2010). LEAP: learning embeddings for atlas propagation. NeuroImage.

[b0340] Yesavage J.A., Brink T.L., Rose T.L., Lum O., Huang V., Adey M., Leirer V.O. (1982). Development and validation of a geriatric depression screening scale: a preliminary report. J. Psychiatr. Res..

[b0345] Zaborszky L., Hoemke L., Mohlberg H., Schleicher A., Amunts K., Zilles K. (2008). Stereotaxic probabilistic maps of the magnocellular cell groups in human basal forebrain. NeuroImage.

