# Supplementary material for: Reduced cortical cholinergic innervation measured using [18F]-FEOBV PET imaging correlates with cognitive decline in mild cognitive impairment
Source: Neuroimage Clin. 2022 Mar 24;34:102992. doi: 10.1016/j.nicl.2022.102992 (PMC8958543; doi:10.1016/j.nicl.2022.102992)
Supplement: Supplementary data 1 [file mmc1.docx]

**Supplementary Information**

| 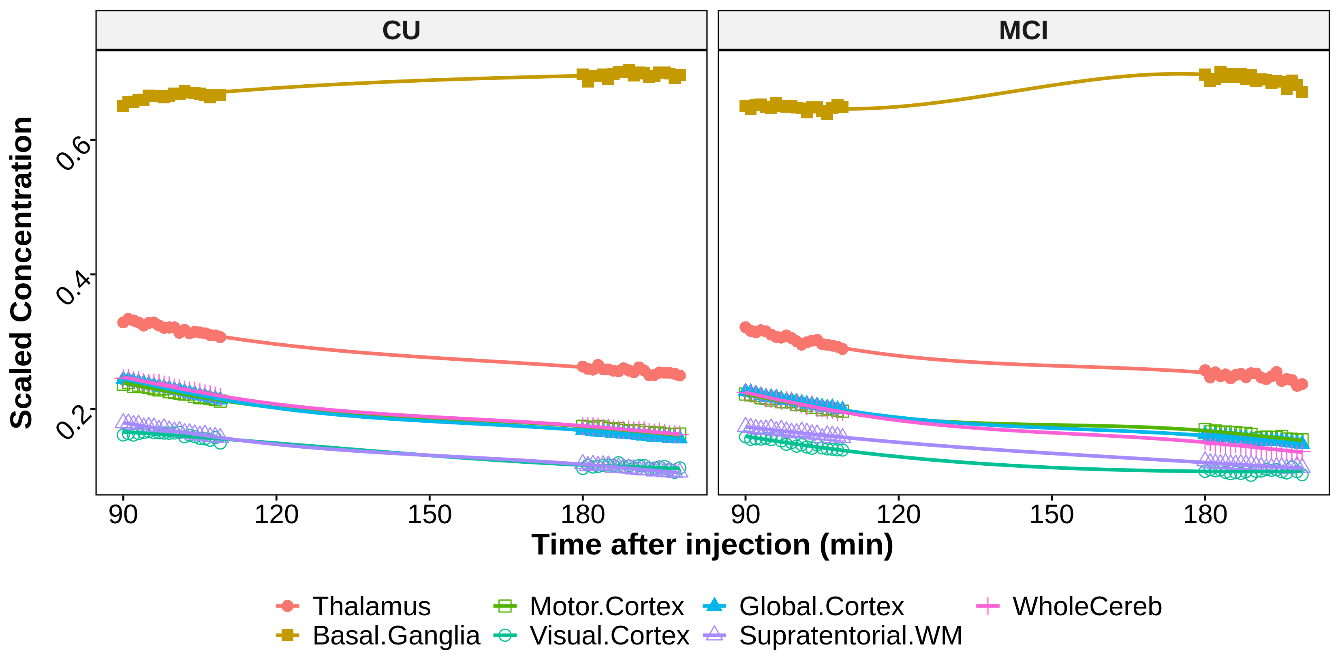 |
| --- |
| **Supplementary Figure 1**. Average tissue time-activity curves in the (left) CU and (right) MCI groups, which demonstrate FEOBV concentrations decaying with time for several brain regions. The FEOBV concentrations were scaled for the injected dose. The supratentorial WM region was used as reference region in this work. |
